# Genomic instability in the *PARK2* locus is associated with Parkinson’s disease

**DOI:** 10.1007/s13353-015-0282-9

**Published:** 2015-04-02

**Authors:** Wojciech Ambroziak, Dariusz Koziorowski, Kinga Duszyc, Paulina Górka-Skoczylas, Anna Potulska-Chromik, Jarosław Sławek, Dorota Hoffman-Zacharska

**Affiliations:** 1 Department of Medical Genetics, Institute of Mother and Child, Kasprzaka 17A, 01-211 Warsaw, Poland; 2 Institute of Genetics and Biotechnology, Faculty of Biology, University of Warsaw, Pawińskiego 5a, 02-106 Warsaw, Poland; 3 Department of Neurology, Faculty of Heath Science, Medical University of Warsaw, Kondratowicza 8, 03-242 Warsaw, Poland; 4 Department of Neurology, Medical University of Warsaw, Banacha 1, 02-097 Warsaw, Poland; 5 Department of Neurological and Psychiatric Nursing, Medical University of Gdańsk, Dębinki 7, 80-952 Gdańsk, Poland

**Keywords:** Common fragile sites, FRA6E, Genomic rearrangements, Parkinson’s disease, *PARK2*

## Abstract

Parkinson’s disease (PD) is a common neurodegenerative disorder affecting mostly elderly people, although there is a group of patients developing so-called early-onset PD (EOPD). Mutations in the *PARK2* gene are a common cause of autosomal recessive EOPD. *PARK2* belongs to the family of extremely large human genes which are often localised in genomic common fragile sites (CFSs) and exhibit gross instability. *PARK2* is located in the centre of FRA6E, the third most mutation-susceptible CFS of the human genome. The gene encompasses a region of 1.3 Mbp and, among its mutations, large rearrangements of single or multiple exons account for around 50 %. We performed an analysis of the *PARK2* gene in a group of 344 PD patients with EOPD and classical form of the disease. Copy number changes were first identified using multiplex ligation probe amplification (MLPA), with their ranges characterised by array comparative genomic hybridisation (aCGH). Exact breakpoints were mapped using direct sequencing. Rearrangements were found in eight subjects, including five deletions and three duplications. Rearrangements were mostly non-recurrent and no repetitive sequences or extended homologies were identified in the regions flanking breakpoint junctions. However, in most cases, 1–3 bp microhomologies were present, strongly suggesting that microhomology-mediated mechanisms, specifically non-homologous end joining (NHEJ) and fork stalling and template switching (FoSTeS)/microhomology-mediated break-induced replication (MMBIR), are predominantly involved in the rearrangement processes in this genomic region.

## Introduction

Parkinson’s disease (PD) (MIM 168600) is the second most common neurodegenerative disorder, of which around 10 % of cases have genetic underpinnings (Thomas and Beal 2007). Early-onset PD (EOPD) is a form of the disease when the first symptoms appear before the age of 45 years. Additionally, juvenile PD (PDJ) is a subdivision of EOPD where the first symptoms appear at 30 years of age or even earlier (Schrag et al. 1998; Friedman 1994). The list of genetic factors causing or increasing the risk of PD has been constantly growing over the last 15 years. However, mutations of three genes, *PARK2* (MIM 602544), *PINK1* (MIM 608309) and *DJ-1* (MIM 602533), are responsible for the majority of EOPD cases following autosomal recessive (AR) pattern of inheritance (Bonifati 2014). *PARK2* gene mutations are particularly common among AR-EOPD patients and the vast majority of PDJ patients are diagnosed with mutations in this gene (Kitada et al. 1998; Lücking et al. 2000). To date, over 200 different *PARK2* mutations have been identified, half of which are point mutations and the other half large gene rearrangements. However, it is still unknown if all the identified genetic changes are pathogenic (Hedrich et al. 2004). The *PARK2* gene is located on chromosome 6, locus 6q25.2-q27 and encompasses a genomic region of 1.3 Mbp. The gene consists of 12 exons and its 4.5 kbp transcript contains a 1,395 bp open reading frame (NCBI 2012). *PARK2* belongs to the family of extremely large human genes and is located within FRA6E, one of the most unstable common fragile sites (CFSs) of the human genome (Smith et al. 2006).

CFSs are intrinsically difficult to replicate genomic regions prone to forming chromosomal breakages (Glover 1998; Sutherland et al. 1998). They are highly conserved and constitute a normal part of chromosomes, although the level of their expression is variable among individuals (Denison et al. 2003a). CFSs are stable in cultured cells, but form gross mutations when exposed to replication stress. Replication inhibitors like aphidicolin, bromodeoxyuridine (BrdU) and 5-azacytidine have been used to induce the expression of CFSs (Durkin and Glover 2007).

CFSs are known to play a major role in carcinogenesis (Smith et al. 2006). They are hotspots for gene amplifications (Coquelle et al. 1997), viral integration (Popescu and DiPaolo 1989) and are also preferentially involved in sister–chromatid exchange (Glover and Stein 1987). It has been reported that the majority of the largest human genes form part of CFSs (Smith et al. 2006). Little is known about direct mechanisms underlying the susceptibility to breakages and rearrangements of CFSs. Nevertheless, some factors have been considered to contribute to instabilities, including late-replicating genomic regions, high AT content, flexible DNA sequences or regions enriched in repetitive elements (Wells and Ashizawa 2006; Ma et al. 2012).

FRA6E is one of 16 CFSs to have been characterised to date at the molecular level (Lukusa and Fryns 2008) and the third most mutation-susceptible of them (Denison et al. 2003b). In cultured cells, its instability can be induced by aphidicolin. The exact size of the region of instability of this CFS is not yet clear; however, it has been suggested that it may span even 9 Mb at 6q25.1-6q26 (Russo et al. 2006). Although FRA6E contains many genes, its main fragility core is localised on the telomeric end, within the *PARK2* gene sequence (Denison et al. 2003b; Palumbo et al. 2010). The mutational hotspot spans the region between exons 2 and 8 of the gene (Denison et al. 2003c).

In order to investigate why CFSs are susceptible to rearrangements, it is necessary to shed light on the clustering and sequence features of breakpoint regions. Among a cohort of Polish PD patients, we identified eight cases of *PARK2* gene rearrangements (deletions and duplications). Their breakpoints have been identified and characterised. Subsequently, we attempted to pinpoint which molecular mechanism is predominantly involved in rearrangement formation in the FRA6E locus.

## Materials and methods

### Subjects

Analysis was performed using the DNA of 344 patients with EOPD and idiopathic, late-onset form of PD (LOPD) who entered the study on the molecular background of PD in patients of Polish origin. The group consisted of 171 EOPD and 173 LOPD patients. Males were a small majority in both of them (EOPD, *n* = 97, 57.30 %; LOPD, *n* = 99, 57.65 %). The age of onset in the entire group varied between 12 and 88 years, among EOPD patients 12 and 45 years [mean 36.8; standard deviation (SD) ± 6.98] and among LOPD patients 46 and 88 years (mean 58.33; SD ± 9.79). At the time of examination, the mean patient age was 55.83 years (SD ± 13.37); in the EOPD and LOPD groups, respectively, it was 45.53 years (SD ± 8.35) and 66.07 years (SD ± 8.76). All patients were assessed by a neurologist and neurological examinations were performed by clinicians experienced in the diagnostic of movement disorders (partial clinical data already published by Koziorowski et al. 2010, 2013; Hoffman-Zacharska et al. 2013). Clinical diagnosis of PD patients was established according to the UK Parkinson’s Disease Brain Bank criteria (Hughes et al. 2001).

Genomic DNA samples of all subjects came from the DNA bank of the Department of Molecular Genetics, Institute of Mother and Child in Warsaw, Poland. DNA of the subjects from the control group belonged to healthy individuals, with no family history of any neurological diseases. All the DNA samples were previously screened for point mutations in all 12 exons of *PARK2* using Sanger sequencing.

All participants signed an informed consent.

The ethic committees of Warsaw Medical University and the Institute of Mother and Child approved the study.

### DNA screening

In order to detect large *PARK2* rearrangements, we employed the multiplex ligation probe amplification (MLPA) technique using commercially available SALSA P051-C1 and P052-C1 Parkinson MLPA kits (MRC-Holland). Both probe sets contain probes specific for all the *PARK2* exons, as well as selected exons of other genes associated with different PD forms (http://www.mrc-holland.com). MLPA experiments were performed according to the manufacturer’s instructions. The probes were hybridised for 14 h and 100 ng of DNA was used. Reaction products were separated with an ABI 3130 Genetic Analyzer (Applied Biosystems). GeneMarker v1.51 software (SoftGenetics LLC) was used for dosage ratio analysis (standard parameters, dosage ratio boundaries <0.75 and >1.25 for deletions and duplications, respectively).

### Rearrangement analysis

To confirm the presence of identified deletions/duplications and to define more precisely the range of rearranged regions, array comparative genomic hybridisation (aCGH) was performed using the NimbleGen 385K chromosome 6 tiling array with 349 bp median probe spacing (NimbleGen, Roche). Arrays were scanned on an Agilent G2565CA Microarray Scanner System (Agilent) and images were analysed using DEVA v1.0.2 software (NimbleGen, Roche). Genomic positions of the rearrangements were specified according to the Human Mar.2006 (NCBI36/hg18) assembly (UCSC Genome Browser, http://genome.ucsc.edu).

### Breakpoint mapping

Based on the coordinates of aCGH probes, for each of the patients, we designed primer pairs using Primer3 software (http://biotools.umassmed.edu/bioapps/primer3_www.cgi). The specificity of oligonucleotides was validated with in-silico polymerase chain reaction (PCR) (UCSC Genome Browser). In order to map the exact breakpoints, we performed PCR reactions using the FastStart High Fidelity PCR System (Roche). PCR products were directly sequenced on both strands with primers used for PCR amplification. Sequencing reactions were performed with BigDye Terminator v3.1 (Applied Biosystems). Electrophoresis was performed on an ABI 3130 Genetic Analyzer (Applied Biosystems). Obtained sequences were analysed in comparison to the reference sequence NM_004562.2.

In two duplication cases (Ex2_5dup and Ex2dup), basic PCR and direct sequencing was unsuccessful; hence, breakpoint sequences were obtained using the inverse PCR method with Mph1103I (Fermentas) and BbvI (New England Biolabs) restriction enzymes, respectively. Genomic DNA was digested, purified and subsequently ligated with T4 DNA ligase (Fermentas). Purified circular DNA fragments encompassing breakpoints were amplified using DreamTaq DNA Polymerase (Thermo Scientific) and sequenced as described previously. Breakpoints identified using this method were then confirmed by sequencing of the native DNA using newly designed sets of primers (all primer sequences and reaction conditions are available upon request).

### Breakpoint region sequence analysis

Sequenced breakpoints of identified rearrangements were subjected to bioinformatics analyses. RepeatMasker (http://www.repeatmasker.org), a program screening DNA sequences for interspersed repeats and low-complexity regions, was used to check for repetitive elements 100 bp upstream and downstream of the breakpoint. Sequence similarity (unrelated to repetitive elements) between 5′ and 3′ breakpoint regions was checked with the ClustalW2 alignment tool (http://www.ebi.ac.uk/Tools/msa/clustalw2). palindrome software (http://emboss.bioinformatics.nl/cgi-bin/emboss/palindrome) search allowed the identification of potential secondary structure-forming sequences around the breakpoint region.

## Results

### Identification of *PARK2* rearrangements

From the cohort of 344 PD patients that was screened for the *PARK2* rearrangements, deletions and duplications encompassing different exons were identified in eight subjects. The *PARK2* PD is characterised by a broad range of clinical phenotypes, sometimes atypical signs, early-onset, slower progression, good response to levodopa, often with more severe dopa-induced complications. *PARK2* mutations carriers revealed clinical features similar to those described earlier. Asymmetrical rest tremor was the dominant symptom at onset. In one case, lower limbs dystonia was the predominant syndrome. Depression and anxiety was the dominant and early symptom in three cases. Response to levodopa treatment was very good for all of them (in one case, it was excellent). Three patients had early fluctuations and dyskinesias (Table 1).

**Table 1:** Clinical and molecular characterisation of the eight Parkinson’s disease (PD) patients with identified *PARK2* deletions and duplications

ID	Age of onset	Age at analysis	*PARK2* mutations		Genotype (cDNA )^a^	Main clinical symptoms	PD type
			Mutation1	Mutation2			
1	32	39	Ex3del	c.1337G > T p.Cys446Phe	c.[172-?_412 + ?del];[1337G > T]	Rigidity, depression and anxiety	EOPD
2	31	40	Ex4_7del	c.101_102del p.Gln34Argfs*5	c.[413-?_871 + ?del(;)101_102del)]	Rest tremor	EOPD
3	26	29	Ex3del	Ex4_7del	c.[172-?_412 + ?del];[ 413-?_871 + ?del]	Bradykinesia, depression and anxiety	EOPD
4	24	33	Ex3_4del	c.101_102del p.Gln34Argfs*5	c.[174-?_534 + ?del];[101_102del]	Dominating lower limbs dystonia	EOPD
5	60	75	Ex6_7del	–	c.[619-?_871 + ?del];[=]	Rest tremor, late fluctuation and dyskinesia	LOPD
6	33	42	Ex2_5dup	c.734A > T p.Lys211Asn	c.[8-?_618 + ?dup];[c.734A > T]	Rest tremor, early fluctuation and dyskinesia, depression, early postural instability	EOPD
7	36	61	Ex2dup	–	c.[8-?_171 + ?dup];[=]	Rigidity, early fluctuation and dyskinesia	EOPD
8	54	49	Ex2dup	–	c.[8-?_171 + ?dup];[=]	Rest tremor, early fluctuation and dyskinesia, excellent response for levodopa treatment	LOPD

^a^HGVS v2 nomenclature, den Dunnen and Antonarakis (2000); reference sequence NM_004562.2 (NCBI37/hg19)

**Fig. 1:**
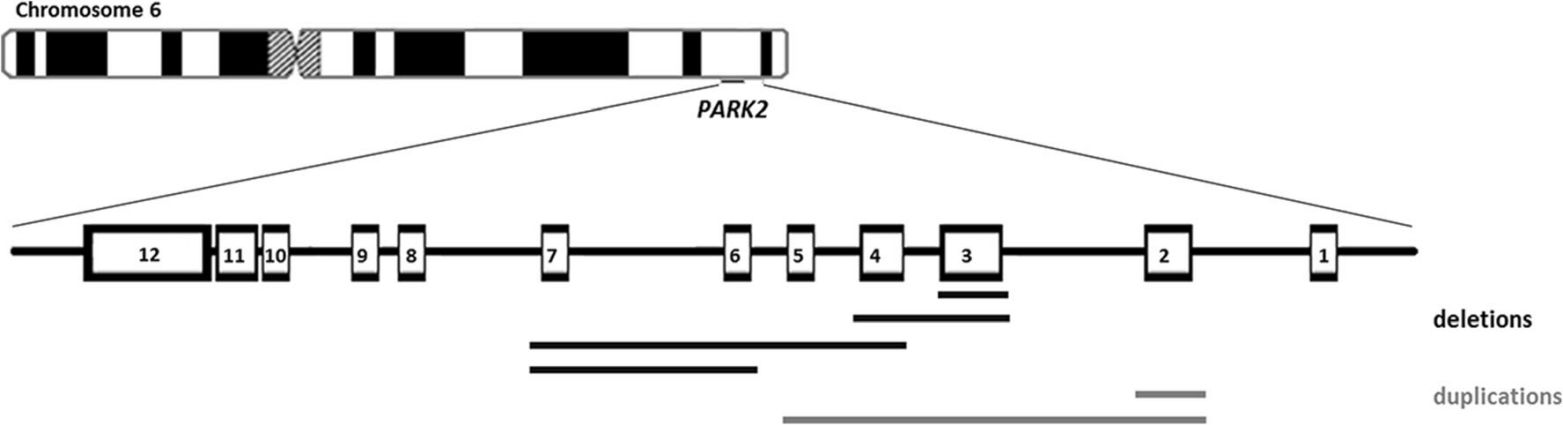
Representation of the ranges of all *PARK2* rearrangements found in the study. Exons of the *PARK2* gene are represented by *numbered boxes*. Identified deletions are depicted in *black* and duplications in *grey*. Rearrangements encompassed from one to four exons in the region between exons 2 and 7. Mutations Ex4_7del and Ex2dup were identified in two subjects

**Fig. 2:**
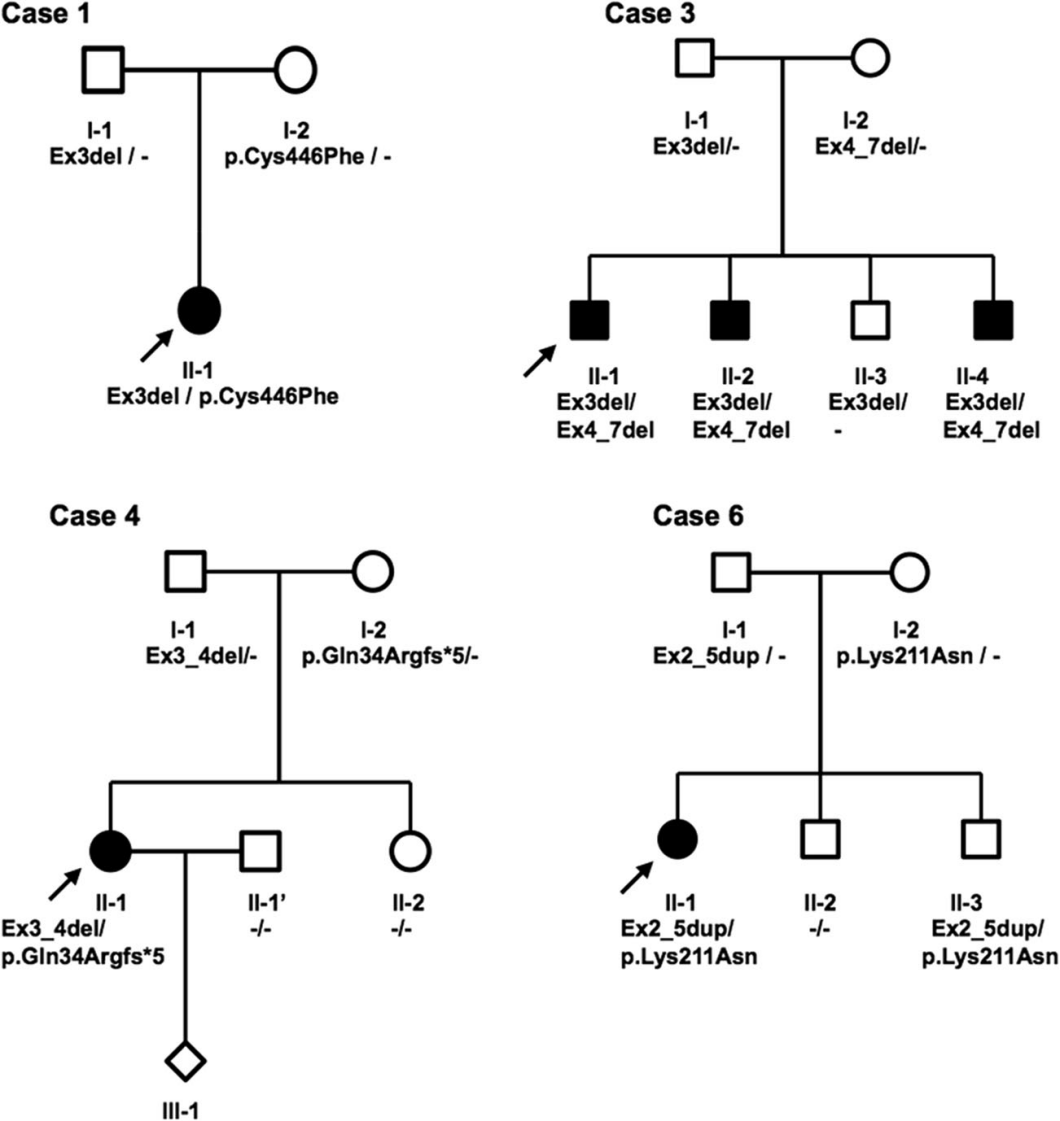
Four early-onset Parkinson’s disease (EOPD) pedigrees showing the *PARK2* mutations’ inheritance in families. *Solid symbols* individuals with EOPD; *open symbols* unaffected individuals without identified mutations, mutations carriers on one or, like in case 6 III-2, on both alleles in the presymptomatic stage at the moment of analysis; index patients are denoted by *arrows*

**Fig. 3:**
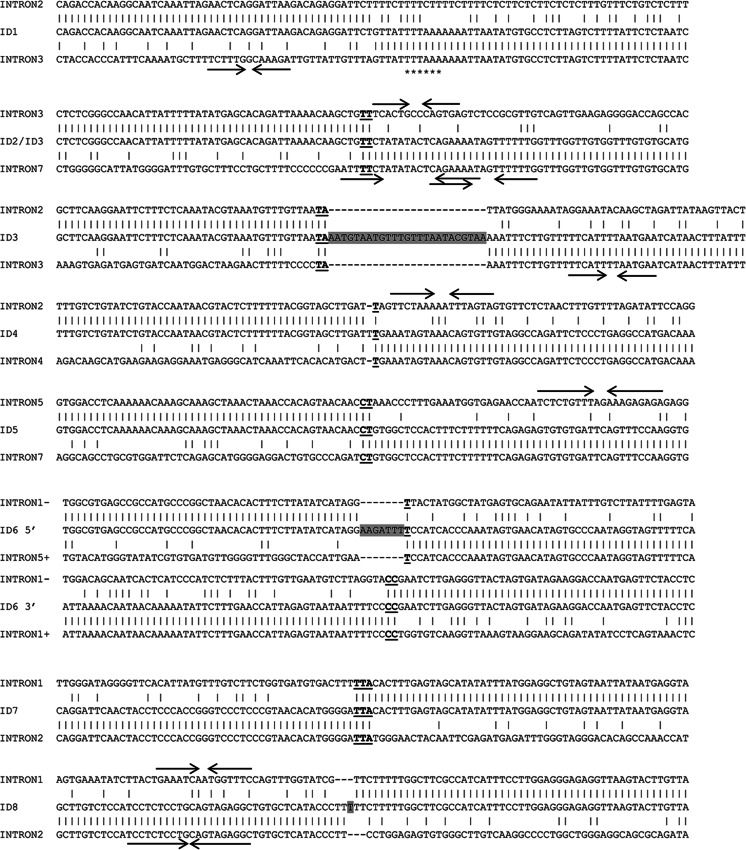
DNA sequences spanning eight identified rearrangements aligned with corresponding normal intronic regions. Homology regions across junctions are **bold and underlined**. The *grey boxes* indicate inserted sequences. The *arrows* indicate palindromic sequences able to form DNA hairpins and the *asterisks* indicate TTTAAA sequence known to be able to induce a curvature in the DNA molecule. 1–3-bp microhomologies were found in 6/8 cases. In the case of subject ID3, both heterozygous deletions (Ex4-7del and Ex3del) are presented. Breakpoints of the Ex4_7del mutation are identical in cases ID2 and ID3. In the case of subject ID6, the duplication is represented by two separate alignments (at the 5′ and 3′ sites) due to an inverted orientation. In cases ID3, ID6 and ID8, the insertions are of unknown origin

The *PARK2* gene rearrangements, five deletions and three duplications, were identified among patients with both EO and LO forms of PD (Fig. 1). Four patients diagnosed with EOPD showed compound heterozygosity, being carriers of one deletion/duplication and a point mutation. Biallelic state of these mutations was confirmed by parental analysis when possible (Table 1, Fig. 2). In one case (EOPD), we were able to prove biallelic deletions only by the analysis of the proband’s parents, as in MLPA they appeared as one continuous deletion. In the remaining three cases (two LOPD and one EOPD), only one heterozygous mutation was identified. Deletions encompassed from one to four exons in the region between the 3rd and 7th exons of the gene. Only one deletion (Ex4_7del) was recurrent and identified in unrelated families (Tables 1 and 2). Duplications in two cases covered only one exon (Ex2dup) and, in one case, four consecutive exons (Ex2_5dup). Analysis of the data obtained from aCGH allowed more detailed characterisation of the identified rearrangements in terms of their range and breakpoint localisation (Table 2). Identification of the breakpoint regions enabled exact mapping of the breakpoints and their analysis for sequence features and similarity. Alignments of 5′ and 3′ sites of the breakpoint regions did not show any long-range homologies in any of the cases. Likewise, no repetitive DNA elements were found on both sides of the breakpoints (100 bp upstream and downstream; Table 2). The Ex4_7 deletion with identical breakpoints was found in two probands. In the case of Ex2_5dup, the mutation was found to be in inverted orientation and contained a triplication of a small fragment of intron 1. Palindromic motifs in close proximity to the breakpoint were found in six cases (seven alleles). The TTTAAA sequence, known for inducing curvature in the DNA, was found in one case. In three cases, short insertions were identified, although their origin is unknown. Subsequently, breakpoint spanning sequences were aligned with normal ones, revealing microhomologies being present at the breakpoint junctions in six cases (7 out of 9 alleles) (Fig. 3).

**Table 2:** Characterisation of the *PARK2* gene rearrangements identified among PD patients

ID	Rearrangement characterisation			Repetitive elements within breakpoint regions (5′/3′)
	MLPA	aCGH Size of del/dup ^a,b^Rearrangements description according to ISCN 2013	Breakpoint coordinates Size of del/dup ^c^Rearrangements description according to HGVS v2	
*PARK2* deletions				
1	Ex3del	16,904 bp arr[hg18] 6q26g.(162,620,085-162,603,181)x1 pat	17,390 bp Chr6(NCBI36):g.162,620,515_162,603,125del	LINE-1/Unique
2	Ex4_7del	483,375 bp arr[hg18] 6q26g.(162,546,840-162,063,483)x1	483,785 bp Chr6(NCBI36):g.162,547,004_162,063,219del	Unique/Unique
3*	Ex3_7del	164,683 bp (Ex3del) arr[hg18] 6q26g.(162,734,637-162,569,954)x1 pat/483,357 bp (Ex4_7del) arr[hg18] 6q26g.(162,546,840-162,063,483)x1 mat	165,563 bp Chr6(NCBI36):g.162,735,128_162,569,565delAATGTAATGTTTGTTTAATACGTAAins/483,785 bp Chr6(NCBI36):g.162,547,004_162,063,219del	Unique/Unique Unique/Unique
4*	Ex3_4del	176,434 bp arr[hg18] 6q26g.(162,691,028-162,514,594)x1 pat	176,803 bp Chr6(NCBI36):g.162,691,457_162,514,654del	LINE-1/Unique
5	Ex6_7del	317,108 bp arr[hg18] 6q26g.(162,382,338-162,065,230)x1	317,810 bp Chr6(NCBI36):g.162,382,573_162,064,763del	Unique/Unique
*PARK2* duplications				
6*	Ex2_5dup	647,223 bp arr[hg18] 6q26g.(163,009,828-162,362,605)x3 pat	646,341 bp Chr6(NCBI36):g.162,362,806_163,009,147dupinvAAGATTTins^d^	SINE/Alu/Unique
7	Ex2dup	115,911 bp arr[hg18] 6q26g.(162,824,231-162,708,320)x3	116,072 bp Chr6(NCBI36):g.162,824,128_162,708,056dup	Unique/LTR/ERVL-MaLR
8*	Ex2dup	199,391 bp arr[hg18] 6q26g.(162,835,867-162,636,476)x3	198,650 bp Chr6(NCBI36):g.162,835,997_162,637,347dupTins	Unique/Unique

*Clinical data and partial genetic data already published in Koziorowski et al. (2010, 2013)

^a^Genomic coordinates according to the distal internal deletion/duplication probes NimbleGen CGH 385K chromosome 6 tiling v2.0D array; NCBI36/hg18 assembly

^b^ISCN 2013 nomenclature, Simons et al. (2013)

^c^HGVS v2 nomenclature, den Dunnen and Antonarakis (2000)

^d^Genomic coordinates according to (+) strand

Several papers have been published so far where exact mutational breakpoints are mapped, but their characteristics on the level of the DNA sequence are rarely discussed. We decided to compare our findings to the data from two recently published articles: Elfferich et al. (2011) and Mitsui et al. (2010). Elfferich et al. submitted *PARK2* breakpoints identified in 13 indexed PD patients (11 deletions and two duplications). Only one of the identified mutations (Ex4del) shared identical breakpoint coordinates with previously reported ones (Table 3). Based on the published coordinates of deletions/duplications identified in that study, we were able to perform sequence analysis of the 5′ and 3′ breakpoint regions for those *PARK2* rearrangements, and found that, in 9 of 13 cases, microhomologies at breakpoint junctions were present (Table 3). Moreover, one of the rearrangements, Ex2dup, had exactly the same breakpoint coordinates as a duplication published in the present study (ID8). Mitsui et al. also published breakpoint analyses of rearrangements identified in a large number of probands of both Asian and European descent. Since we did not find coinciding breakpoints between these two groups, only cases of European descent were taken into consideration. We found that an identical Ex2dup mutation, identified in Elfferich et al. and in the present study, was also identified by Mitsui et al. Similarly, the majority of cases reported by them contained microhomologies at breakpoint junctions. In addition, a great majority of the rearrangements identified in both of the mentioned studies fell into the region between the 2nd and 8th exons of the *PARK2* gene (Table 3).

**Table 3:** Summary of breakpoint characteristics of the rearrangements identified in the present and two previous studies*

Mutation	Breakpoint region homology (5′/3′)	Microhomologies at breakpoint junctions	Number of cases	Source
*PARK2* deletions				
Ex1del	–	+ (ATCT)	1	Elfferich et al. (2011)
Ex2delA	–	+ (AG)	1	Elfferich et al. (2011)
Ex2delB	–	+ (CCTGA)	1	Elfferich et al. (2011)
Ex2delC	–	+ (T)	1	Elfferich et al. (2011)
Ex3del	–	–	1	Present study
Ex3del	–	+ (TA)	1	Present study
Ex3delA	–	+ (T)	1	Elfferich et al. (2011)
Ex3delB	–	+ (CT)	2	Elfferich et al. (2011)
Ex3del	–	+ (CT)	2	Mitsui et al. (2010)
Ex3_4del	–	+ (T)	1	Present study
Ex3_5del	–	–	1	Elfferich et al. (2011)
Ex3_6del	–	+ (GAT)	2	Mitsui et al. (2010)
Ex4del	–	–	2	Elfferich et al. (2011)^a^
Ex4del	–	+ (AGCAC)	3	Mitsui et al. (2010)
Ex4_7del	–	+ (TT)	2	Present study
Ex5del	–	+ (T)	1	Elfferich et al. (2011)
Ex5_6del	–	+ (CC)	3	Elfferich et al. (2011)
Ex6_7del	–	+ (CT)	1	Present study
Ex8_10del	–	–	1	Elfferich et al. (2011)
*PARK2* duplications				
Ex2dup	–	+ (TTA)	1	Present study
Ex2dup	–	–	4	Present study, Elfferich et al. (2011), Mitsui et al. (2010)
Ex2_5dup + inv	–	+ (T)/+ (CC)	1	Present study
Ex7dup	SINE/Alu/ SINE/Alu	–	4	Elfferich et al. (2011)

*From Mitsui et al. (2010); only cases with exon-spanning rearrangements of European descent were taken into consideration

^a^Two cases also previously published by Hedrich et al. (2004)

## Discussion

Since the gene’s discovery in 1998, a broad range of mutations in *PARK2* has been identified. Abnormalities have been found in all 12 exons of the gene, although a majority of them fall into the region between exons 2 and 8 (Denison et al. 2003c). Around 50 % of the mutations are large rearrangements of one or more exons (Hedrich et al. 2004), which, in heterozygous configuration, are undetectable by traditional Sanger sequencing. In our study, we have identified large rearrangements in 8 of the 344 PD patients examined (2.33 % of patients, 1.31 % of alleles). Identified rearrangements span the regions between exons 2 and 8, which is in accordance with the fragility centre of the FRA6E. However, most of the mutations were unique, without common breakpoints. Only in two cases, with Ex4_7del, were the breakpoints identical. These two subjects are unrelated, to our knowledge. However, the kinship could not be completely excluded, meaning that there may be a common founder for this mutation, especially that no common recombination susceptibility has been found in this region.

Based on case ID3, showing the continuous deletion of exons from 3 to 7 in the MLPA analysis, we conclude that, in terms of molecular diagnostics, it is important not to rely exclusively on this method when identifying genomic rearrangements (Table 2). MLPA is convenient and robust to simultaneously screen many genomic samples, but in some instances, significant information on the biallelic nature of the mutation might be missed using only this technique. Therefore, it is always necessary to check the parental state of identified mutation(s), if possible. Alternatively, more precise techniques, like aCGH or specific PCR (Elfferich et al. 2011), may be used.

In some cases, of not only idiopathic but also EOPD, only one heterozygous mutation is identified in *PARK2* or other AR-PD-associated genes (cases ID5, ID7 and ID8, Table 1), which is difficult to interpret. However, it is possible that the second mutation is localised in one of the regulatory elements, like promoters, enhancers or regulatory non-coding RNAs, which is still beyond our detection capabilities. On the other hand, it is plausible that heterozygous mutations in one of such genes, juxtaposed with some other genetic variants, are sufficient to cause the incidence of a disease or increase the risk of its occurrence.

Genomic rearrangements may be brought about by various mechanisms. The most common cause of genomic rearrangements are recombination events between two DNA blocks of high homology, like low-copy repeats (LCRs), LINE or SINE elements, via non-allelic homologous recombination (NAHR) (Stankiewicz and Lupski 2002). NAHR generates recurrent rearrangements, that can be observed in the same shape in unrelated individuals, and may occur during both mitosis and meiosis (Lam and Jeffreys 2006; Darai-Ramqvist et al. 2008). However, despite the high density of various repetitive elements in the intronic sequences of the *PARK2* gene in general, the breakpoint regions identified did not contain homologous sequences upstream and downstream of the breakpoint in any of cases. It means that recombination events are not likely to have been responsible for generating identified rearrangements. Non-recurrent rearrangements may be driven by more than just one mechanism. Two of the most prevalent mechanisms that may generate large rearrangements are non-homologous end joining (NHEJ) and microhomology-mediated break-induced replication (MMBIR).

NHEJ is a major cellular mechanism for double-strand break (DSB) repair (Moore and Haber 1996). Upon DSB, NHEJ reconnects chromosome ends, very often editing them before ligation, thus leaving so-called an information scar (Lieber 2008). While editing NHEJ is able to add random nucleotides at the site of a breakage to facilitate the strands’ alignment, which often occurs based on short homologies, and ligation (Labhart 1999), NHEJ in most instances causes deletions, but, in combination with homologous recombination (HR), may also be responsible for duplications (Woodward et al. 2005).

Another mechanism proposed to trigger non-recurrent genomic rearrangements is fork stalling and template switching (FoSTeS), a mechanism that was originally proposed to explain multiple duplications found in Pelizaeus–Merzbacher disease patients, and that has been later generalised as MMBIR (Lee et al. 2007; Zhang et al. 2009). FoSTeS/MMBIR is a microhomology-based mechanism that can explain very complex rearrangements, both deletions and duplications. It utilises microhomologies to switch the template upon replication fork stalling and collapse. Replication continues based on a wrong template until the original fork is restored. The template being switched to is usually in physical proximity but not necessarily in close linear proximity to the original replication fork. Such template switching may occur several times before the replication process gets back to its original template, resulting in complex rearrangements (Lee et al. 2007).

**Fig. 4:**
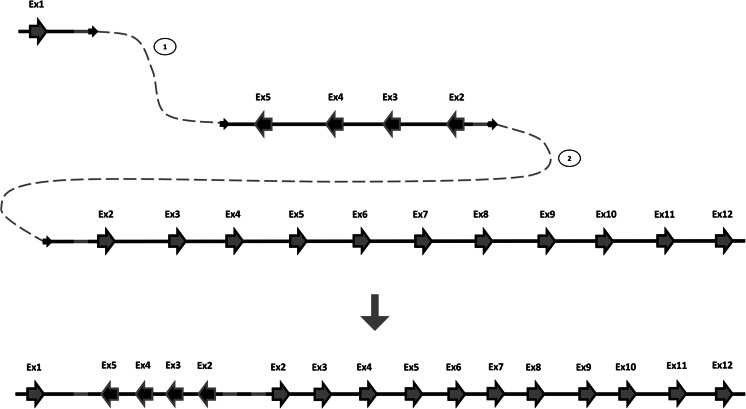
Predicted fork stalling and template switching (FoSTeS)/microhomology-mediated break-induced replication (MMBIR) mechanism of the Ex2-5dup mutation in subject ID6. Upon replicating, the first exon of the *PARK2* gene replication fork stalled and one strand invaded either the sister molecule or the homologue chromosome in inverted orientation (*1*), resulting in inverted duplication. Subsequently, the original forks were restored, but primed upstream of the point where it first stalled (*2*), resulting in triplication of the grey-highlighted region

As no extended homologies or repetitive elements were found within the sequences encompassing the breakpoints (Table 2), the occurrence of homologous recombination-based mechanisms can be excluded. Alignments of the breakpoint regions identified in our study with normal genomic sequences reveal that 7 out of 9 breakpoint junctions contain 1–3-bp homologies (Fig. 3). Most of the breakpoint regions identified contained palindromic sequences which, upon the spontaneous formation of stable secondary DNA structure, may lead to replication fork stalling or cause a DSB. Only one breakpoint region contains the TTTAAA sequence, known to be able to induce a curvature in the DNA molecule (Trifonov 1985). Both palindromic and TTTAAA motifs are potentially involved in DSB events. These findings strongly support microhomology-mediated mechanisms following replication fork collapses, predominantly NHEJ and FoSTeS/MMBIR, as major mechanisms responsible for genomic rearrangements within the *PARK2* gene, the fragility centre of FRA6E. An illustration of the FoSTeS/MMBIR rearrangement mechanism, based on the case ID6, is shown in Fig. 4. Additionally, analysis of sequences around breakpoints published in previous studies, where the majority of identified *PARK2* rearrangements also contained microhomologies at the junctions, supports our hypothesis (Table 3).

The data on *PARK2* rearrangements have been published previously (Hedrich et al. 2004; Clarimon et al. 2005; Nakaso et al. 2006; Bayrakli et al. 2007; Mitsui et al. 2010; Elfferich et al. 2011). Our findings stay in concordance with these reports; the majority of rearrangements identified in the *PARK2* gene fall into the region between exons 2 and 8 (Tables 2 and 3).

The remaining question is why the FRA6E locus, as well as other CFSs in the human genome, is so vulnerable to chromosomal breakages. Studies on *PARK2* mutations reveal that, in spite of the abundance of repetitive elements within the gene sequence, homologous recombination is not a frequent mechanism generating rearrangements. Thus, the direct cause must be rooted in other features of the sequence that delay the replication processes by hindering replication forks’ progress and make them more likely to collapse. It seems reasonable to assume that the presence of palindromic sequences alone is not responsible for this vulnerability, as such sequences are found in many other regions of the genome. However, they may be a facilitating factor for delaying the replication. First studies to address the question of CFSs instability suggested that late-replicating regions confer the susceptibility to rearrangements (Laird et al. 1987; Durkin and Glover 2007). However, CFSs are not the only regions of the human genome that undergo late replication. Recently, it has been found that breakages occur preferentially at the transition zones between sequences undergoing late and early replication (El Achkar et al. 2005; Palumbo et al. 2010). FRA6E contains two such zones, one being located within the sequence of intron 8 of *PARK2*. Nonetheless, whether this is the primary reason why chromosomes break at CFSs is still to be elucidated.
